# Bioaccessibility of metals in food supplements: protocols and facts

**DOI:** 10.1007/s10534-026-00844-4

**Published:** 2026-06-30

**Authors:** Thaís dos Santos Berón, Gustavo Rossato Bitencourt, Rochele Sogari Picoloto, Rodrigo Cordeiro Bolzan, Paola Azevedo Mello

**Affiliations:** Departamento de Química, Universidade Federal de Santa Maria, Av. Roraima, 1000, Santa Maria, RS 97105-900 Brazil

**Keywords:** Food supplements, Essential elements, Bioaccessibility, Unified BARGE method, INFOGEST method, ICP OES

## Abstract

This study evaluated the bioaccessibility of essential (Ca, Cu, Fe, K, Mg, Mn, Mo, Na and Zn) and non-essential (Al, Ba, Cr, Sr and V) elements in food supplements (vitamins and/or minerals and botanicals). Extraction using in-vitro methods (Unified BARGE Method—UBM and INFOGEST) was evaluated allowing a comparison of protocols and using inductively coupled plasma optical emission spectrometry (ICP OES). Bioaccessibility values revealed differences among the elements in different types of food supplements. The limits of quantification (LOQs) obtained for some elements were relatively high for both methods due to the composition of the digests, which drastically impaired bioaccessibility assessment. As a result, most elements presented fractions lower than the LOQ, regardless of the sample. This is an important limitation promoted by interferences when analyzing these extracts by ICP-based techniques. A simple extraction using 1 mol L^−1^ HCl was evaluated, considering that HCl is present in the solutions used in both protocols. Leaching was observed for the same elements using only the HCl solution.

**Supplementary Information:**

The online version contains supplementary material available at 10.1007/s10534-026-00844-4.

## Introduction

The consumption of food supplements has substantially increased worldwide, under the promise of a better quality of life, disease prevention, and esthetics improvement when combined with a balanced diet and physical activity (Camire and Kantor 1999). In Brazil, a 10% increase in the use of food supplements was observed between 2015 and 2020, impacting around 60% of households (ABIAD 2020).

The USA Food and Drug Administration defines food supplements as products to complement the intake of essential nutrients for humans, such as vitamins, minerals, and amino acids (FDA 1994). Among them, special attention should be given to essential and non-essential elements. While the biological role of essential elements in human organisms is well established, influence of non-essential elements remains unknown or inconclusive, despite the research in this area (Chellan and Sadler 2015). Regardless of their essentiality, knowledge about the total concentration of the elements present in food supplements (which depends on the type of food supplement and its manufacturing process) is crucial to ensure an adequate supply to consumers (Smichowski and Londonio 2018). On the other hand, the amount of an element ingested through food supplements (or any food) is often not entirely absorbed by the body, and its actual beneficial or harmful effect on humans cannot be assessed. Hence, not only is the total content important but it is necessary to know the bioaccessibility of an element from a particular food (Ferreira and Tarley 2020; Xu et al. 2024; Hur et al. 2011; Hu et al. 2013).

Oral bioaccessibility is defined as the fraction of an ingested compound or element that is released in the gastrointestinal tract, making it available for absorption. This fraction depends on various factors, including the food matrix, the elemental species present, and the absorption and metabolism of the compound in the body (Galanakis 2021). Some *in vitro* and in vivo protocols have been used to assess the bioaccessibility of elements (Moreda-Piñeiro et al. 2011; Wragg et al. 2011). Depending on the case, *in vitro* protocols are preferred to in vivo ones because of their lower cost and simpler infrastructure required, easier and faster handling, and the absence of ethical issues. In addition, they enable the control of critical experimental conditions (pH, temperature, and time) and the collection of aliquots at different stages of the digestion process (Tokalioğlu et al. 2014; Fernández-García et al. 2009).

The unified Bioaccessibility Research Group of Europe (BARGE) method (UBM, currently an ISO standardized method for soil—ISO 17924) and the INFOGEST method have been widely explored as *in vitro* digestion methods for assessing the bioaccessibility of elements (ISO 17924 2018). Although designed and in vivo validated for determining the bioaccessibility of As, Cd, and Pb in contaminated soils, the UBM method has also been used to digest food matrices including multivitamins and mineral food supplements (Wragg et al. 2011; Tokalioğlu et al. 2014; Denys et al. 2012; Ma et al. 2024). On the other hand, INFOGEST was developed for food (Brodkorb et al. 2019), and has been used as a method for bioaccessibility assessment of, for instance, As and Cd in rice (Ma et al. 2024), nutrients in infant purees (Silva et al. 2018), and microelements in multivitamin supplements (Latif et al. 2025). Considering the operating conditions for each protocol, such as the extraction parameters and the fluid composition, some variations were observed in bioaccessibility values assessed by different methods. The variability in the bioaccessibility of metals assessed by the UBM, Physiologically Based Extraction Test (PBET), by the Solubility Bioaccessibility Research Consortium (SBRC), by the Deutsches Institut für Normung (DIN), and by In Vitro Gastrointestinal (IVG) methods was studied for soils (Yin et al. 2016). Considering food matrices, the comparison of methods was only carried out for vegetables (UBM and PBET) (Wang et al. 2019) and rice (Wang et al. 2020). Additionally, there is only one study comparing the results obtained by the UBM and INFOGEST methods (in addition to PBET) for rice (Ma et al. 2024). This study compared three *in vitro* methods—namely INFOGEST, PBET, and UBM—to assess the bioaccessibility of As and Cd in rice samples. Results showed that intestinal bioaccessibility was generally similar across the three methods, with a significant difference between INFOGEST and UBM for As. A moderate correlation was observed between PBET and INFOGEST values for As (R^2^ = 0.416), and a strong correlation between PBET and UBM values for Cd (R^2^ = 0.879). Additionally, PBET indicated that polished rice had 17.0% and 19.8% higher bioaccessibility of As and Cd, respectively, compared to unpolished rice. The study emphasizes the impact of *in vitro* methods and rice types on bioaccessibility results, highlighting the need for more accurate health risk assessment related to rice consumption (Ma et al. 2024).

To mimic the human gastrointestinal tract, all *in vitro* digestion methods require large quantities of chemical and biological reagents, thus impairing accuracy in analytical techniques for elemental determination and also results in large waste generation. Inductively coupled plasma optical emission spectrometry (ICP OES) has been chosen for elemental determination in many matrices with suitable limits of quantification (LOQs) for several analytes, including food applications (Krejčová et al. 2007; Pecev-Marinkovic´ et al. 2024). Commonly, wet digestion is carried out to promote the decomposition of compounds from sample matrices using concentrated acids (like HNO_3_ and combinations with H_2_O_2_ and/or HCl). This can be a suitable approach for total element determination even though care should be taken to circumvent the well documented spectral and non-spectral interferences (Pecev-Marinkovic´ et al. 2024; Pohl 2007). Contrarily to the solutions obtained through wet acid digestion for total element determination, digestates by bioaccessibility protocols will contain dissolved organic compounds and relatively high content of some elements that are present in the solutions used to mimic the human gastrointestinal tract, impairing the determination step and requiring systematic studies on the protocols, their scope, limitations, and suitability.

Those drawbacks have resulted in studies in which bioaccessibility is estimated by using single-extraction methods, mainly with diluted acids (HNO_3_ or HCl) and metal chelating agents (*e.g.*, EDTA and DTPA) (Pelfrêne et al. 2017, 2020). To the best of our knowledge, bioaccessibility assessment using single-extraction methods has only been tested on soil samples, and no studies encompassing food supplements (or any other food matrix) have been found.

Therefore, the objective of the present work was to evaluate and compare the bioaccessibility of essential (Ca, Co, Cu, Fe, K, Mg, Mn, Mo, Na, Ni, Zn, and V) and non-essential elements (Al, As, Ba, Be, Cd, Cr, La, Pb, and Sr) in food supplements assessed by using the UBM and INFOGEST methods. Inductively coupled plasma optical spectrometry (ICP OES) was used for metals determination in digestates. In addition, metals leaching was evaluated through a simple extraction method, using 1 mol L^−1^ HCl.

## Materials and methods

### Equipment

Bioaccessibility protocols were carried out using an incubator (TE-420, Tecnal Equipamentos Científicos, Brazil) and 150 mL beakers, equipped with an analog temperature controller and a controlled agitation system. The temperature was maintained at 37 ± 2 °C with constant agitation at 200 rpm. A centrifuge (3K30, Sigma, USA) was used in all experiments. All weighing was carried out using an analytical balance (AY220, Shimadzu, Japan) with a resolution of 0.0001 g, or a microbalance (M2P, Sartorius, Germany) with a resolution of 0.001 mg.

To determine the total concentration, the previous digestion of food supplement samples was performed using a microwave oven (ETHOS™ EASY, Milestone, Italy). This instrument was equipped with a rotor capable of holding forty-four PTFE vessels with an individual capacity of 100 mL. The maximum temperature, pressure, and microwave power were 220 °C, 35 bar, and 1800 W, respectively.

The elemental determination in food supplements was carried out using an inductively coupled plasma optical emission spectrometer (ICP OES, Spectro Ciros CCD, Spectro Analytical Instruments, Germany). The instrument was equipped with a double-pass nebulization chamber (Scott-type, Spectro Analytical Instruments) and a quartz torch with a 2.5 mm diameter quartz injector tube (Spectro EOP Quartz Torch, Glass Expansion, Australia). Argon was used (99.996%, White Martins, Brazil) for plasma generation. The operational conditions of the equipment are in Table S1 (Supplementary Material).

### Samples

In this study, four samples of food supplements from different types and compositions were used. The characteristics of each sample are described in Table 1. The samples were acquired in the local market or through e-commerce, originating from both Brazil and the United States. They were coded according to their type (VM for vitamins and minerals, BT for botanicals, and MN for minerals). It is important to note that the absence of information regarding the amino acid linked to the minerals limits any deeper discussion on the related aspects. All samples were dried in an oven for 4 h at 60 °C and stored for further experiments. The samples in tablet form were ground using a mortar and pestle, while capsule samples were opened, and the internal content was used. Sample VM-I was sieved using a mesh with 35 threads (porosity less than 200 µm) due to the tablet coating.

**Table 1:** Information on the samples used in this work

Code	Classification	Label information	Form	Origin
VM-I	Vitamins and minerals	Vitamins A, B1, B2, B3, B5, B6, B7, B9, B12, C, D3, and K, MgO, Zn, Mn, Cu, Se, Mo, Cr, talc, and SiO_2_	Tablet	Brazil
VM-II	Vitamins and minerals	Vitamins A, B1, B2, B5, B6, B7, B9, B12, C, D3, E, and K, CaCO_3_, C_4_H_2_FeO_4_, KI, MgO, ZnO, Na_2_Se, CuSO_4_, MgSO_4_, CrCl_3_, Na_2_MoO_4_, cellulose, modified starch, maltodextrin, sodium croscarmellose, hypromellose, magnesium stearate, SiO_2_, gelatin, and polyethylene glycol	Tablet	USA
BT-I	Botanical	Guarana (*Paullinia cupana*) and açaí (*Euterpe oleracea*) flavoring, catuaba (*Anemopaegma mirandum*), cocoa (*Theobroma cacao*), and ginseng (*Panax ginseng*) extracts, vitamins A, B1, B2, B3, B5, B6, B9, B12, C, E and K2, Cu, Fe, Cr, Mn, Mo, Se, and Zn	Capsule	Brazil
MN-I	Minerals	Calcium amino acid chelate, KH_2_PO_4_, KI, magnesium amino acid chelate, zinc amino acid chelate, C_5_H_11_NO_2_Se, copper amino acid chelate, manganese amino acid chelate, Cr(C_6_H_4_NO_2_)_3_, Mo, KCl, H_3_BO_3_, vanadium amino acid chelate, glutamic acid, rice protein, parsley leaf, alfalfa leaf, horsetail, watercress, dandelion root, yellow dock root, and chamomile	Capsule	USA

*VM* Vitamin and mineral, *BT* Botanical, *MN* Mineral

### Reagents and standards

Water used in the procedures throughout this study was distilled, deionized through an ion exchange column, and purified (Milli-Q® system, Merck Millipore Corp, USA) with a minimum resistivity of 18.2 MΩ cm. Nitric acid (65%, Merck, Germany) and hydrochloric acid (37%, Merck, Germany) used for sample digestion were previously distilled using a sub-boiling system (duoPUR®, model 2.01, Milestone, Italy).

The following reagents were used for bioaccessibility protocols: potassium chloride (Merck, Germany), sodium phosphate (Labsynth, Brazil), potassium thiocyanate (Merck, Germany), sodium sulfate (Sigma Aldrich, USA), sodium chloride (Vetec, Brazil), sodium hydroxide (Vetec, Brazil), calcium chloride (CRQ Química, Brazil), ammonium chloride (Merck, Germany), sodium bicarbonate (Merck, Germany), monopotassium phosphate (Labsynth, Brazil), magnesium chloride (Labsynth, Brazil), urea (Labsynth, Brazil), mucin (Sigma Aldrich, USA), glucose (Proquimica, Brazil), glucuronic acid (Sigma Aldrich, USA), glucosamine (Sigma Aldrich, USA), uric acid (Merck, Germany), α-amylase (A9857, Sigma Aldrich, USA), bovine serum albumin (A7906, Sigma Aldrich, USA), pepsin (porcine, P7000, Sigma Aldrich, USA), pancreatin (porcine, P7545, Sigma Aldrich, USA), lipase (porcine, L3126, Sigma Aldrich, USA), and bovine bile (B3883, Sigma Aldrich, USA).

Calibration curves for elemental determination by ICP OES were prepared in two concentration ranges (1 to 100 µg L^−1^ and 0.25 to 10 mg L^−1^ in 5% v v^−1^ HNO_3_) using standard stock solutions, containing 10 mg L^−1^ (SCP33MS, SCP Science, Canada) and 1000 mg L^−1^ of the analytes (Merck, Germany), respectively.

### Determination of total concentration of essential and non-essential elements in food supplements

Analytes were determined by ICP OES after previous wet acid digestion. A procedure previously applied for food supplement samples was used (Mello et al. 2023). For this, 250 mg of the sample was weighed and inserted into the digestion vessel. Then, 6 mL of concentrated HNO_3_ was added. The vessels were capped, inserted into the rotor, and placed into the microwave cavity. The heating program has a 20 min ramp up to 180 °C and a 20 min holding step, followed by a cooling step (35 min). At the end, the digests were cooled down, transferred to volumetric flasks, and diluted to 25 mL with ultrapure water for further determination of essential and non-essential elements by ICP OES.

### Bioaccessibility of essential and non-essential elements in food supplements

Two *in vitro* methods for determining the bioaccessibility of essential and non-essential elements in food supplements were evaluated and compared: the UBM and the INFOGEST. The results were assessed in terms of bioaccessibility percentages, calculated according to Eq. 1. It is noteworthy to note that five sample replicates and three analytical blanks were performed for all extractions using both methods.

1 $${\mathrm{Bioaccessibility}} \left( {\% {\mathrm{w\,w}}^{ - 1} } \right) = \frac{{{\mathrm{Bioaccessible}}\;{\mathrm{fraction}} (\upmu {\text{g g}}^{ - 1} )}}{{{\mathrm{Total}}\;{\mathrm{concentration}} (\upmu {\text{g g}}^{ - 1} )}} \times 100$$

The bioaccessible fraction was the content (µg g^−1^) determined by ICP OES after extraction through the bioaccessibility protocol and the total concentration was that determined by ICP OES after wet acid digestion (according to 2.4).

#### Unified BARGE method (UBM)

The UBM protocol was carried out based on previous works (Wragg et al. 2011; Tokalioğlu et al. 2014). The composition of each digestive fluid is described in Table S2. This method consists of two stages, resulting in two final extracts: (i) the digested fraction obtained in the first phase (denominated gastric phase) constitutes an extraction solution simulating saliva followed by gastric fluid, and (ii) the digested fraction obtained in the second phase (denominated gastrointestinal phase) constitute an extraction solution simulating the entire gastrointestinal system (saliva + gastric fluid + duodenal fluid + bile). The total digestion time was approximately 2 h.

Approximately 0.3 g of the sample was weighed directly into 50 mL tubes. Then, 4.5 mL of saliva were added followed by manual shaking for 1 min. Subsequently, 6.75 mL of gastric fluid were added, and the tubes were placed in the shaker at 37 ± 2 °C and 200 rpm, for 1 h. After this step, the pH was measured(if it was out of 1.2 to 1.7 range, adjustment was necessary). Afterwards, the extracts were centrifuged at 3000 rpm for 5 min. At the end of this step, an aliquot of 1 mL of the supernatant (gastric phase) was collected, and combined with 9 mL of 0.1 mol L^−1^ HNO_3_ for element determination by ICP OES.

Posteriorly, the gastrointestinal extraction was conducted using the portion that remained in the first stage. In this step, 13.5 mL of duodenal fluid and 4.5 mL of bile were added to the gastric solution. The tubes were manually agitated for 1 min and heated in the shaker at 37 ± 2 °C and 200 rpm for 1 h. After this step, the pH was measured (if it was out of the 6.3 ± 0.5 range, adjustment was necessary). The extracts were then centrifuged at 3000 rpm, for 5 min. Subsequently, a 1 mL aliquot of the supernatant (gastrointestinal phase) was pipetted and combined with 9 mL of 0.1 mol L^−1^ HNO_3_ for element determination by ICP OES.

#### INFOGEST method

The INFOGEST protocol was conducted based on previous work (Brodkorb et al. 2019). Unlike the UBM method, the INFOGEST method is carried out in a single digestion step, covering saliva, gastric fluid, and intestinal fluid (compositions are also shown in Table S2). The enzyme activity was taken as specified by the manufacturers. The total digestion time was about 4 h, conducted at 37 °C and agitation at 200 rpm (Silva et al. 2018).

For these experiments, 5 g of the sample were weighed into 50 mL tubes, and then 4 mL of saliva and 1 mL of CaCl_2_ (7.5 mmol L^−1^) were added. The vials were placed in a shaker with agitation for 2 min. After the first incubation, 9.1 mL of gastric fluid and 0.7 mL of CaCl_2_ (2 mmol L^–^^1^) were added to the vials and shaken for 2 h. Finally, 18.5 mL of duodenal fluid and 1.35 mL of CaCl_2_ (9 mmol L^−1^) were added and the vials were heated at 37 °C for 2 h, with agitation at 200 rpm. After the final incubation step, the extracts were centrifuged at 3000 rpm for 30 min and filtered for analyte determination by ICP OES.

### Extraction with diluted HCl for elemental leaching

Extraction of essential and non-essential elements in food supplements was evaluated using only a 1 mol L^−1^ HCl solution in order to evaluate the effect of leaching on the results. For this, the extraction method was based on the INFOGEST method parameters, *i.e.*, sample mass (5 g), temperature (37 °C), and extraction time (4 h) but the only reagent used was diluted HCl. Final extracts were centrifuged and filtered for ICP OES determination.

## Results and discussion

### Total concentration of essential and non-essential elements in food supplements by ICP OES

The elemental concentration in dietary supplements can vary in a wide range due to several factors, including the chemical form of the element, the specific objectives of the supplement, regulatory standards, bioavailability, formulation, market preferences, ingredient costs, and specific dietary requirements (Bawiec et al. 2025; Devarshi et al. 2024; EFSA, 2024; Dickinson and MacKay 2014). The total concentration of essential and non-essential elements in the food supplement samples evaluated in this study is shown in Table 2. The concentration of Be, Co, Cd, La, Ni, and Pb in all samples was below the LOQ, and they were therefore no longer evaluated in the study. Further research is necessary to determine the total concentration and bioaccessibility of these elements, especially those considered toxic (e.g., Cd and Pb), using more sensitive analytical techniques, such as inductively coupled plasma mass spectrometry (ICP-MS).

**Table 2:** Total concentration of essential and non-essential elements in food supplements by ICP OES (mean ± standard deviation, n = 3)

Element(µg g^−1^)	Sample			
	VM-I	VM-II	BT-I	MN-I
Al^a^	0.201 ± 0.002	0.083 ± 0.002	0.459 ± 0.005	0.074 ± 0.003
Ba	26.6 ± 0.6	1.79 ± 0.02	31.3 ± 0.6	33.3 ± 3.1
Be	< 0.365	< 0.445	< 0.025	< 0.012
Ca^a^	89.7 ± 1.2	161 ± 3	25.6 ± 0.6	261 ± 4
Co	< 62.6	< 78.2	< 41.7	< 96.2
Cu	521 ± 7	1481 ± 81	8.92 ± 1.42	105 ± 8
Cd	< 4.36	< 5.45	< 2.90	< 6.70
Cr	24.3 ± 1.1	91.8 ± 3.2	1.76 ± 0.06	15.6 ± 0.8
Fe^a^	0.206 ± 0.010	10.3 ± 0.1	0.797 ± 0.025	0.151 ± 0.004
K^a^	0.259 ± 0.018	0.122 ± 0.002	10.4 ± 2.2	13.7 ± 0.9
La	< 0.552	< 0.690	< 0.368	< 0.850
Mg^a^	68.5 ± 1.8	66.8 ± 4.3	2.94 ± 0.03	97.5 ± 3.8
Mn^a^	1.64 ± 0.01	2.90 ± 0.11	0.034 ± 0.001	0.783 ± 0.067
Mo	43.2 ± 0.7	61.9 ± 5.0	1.29 ± 0.11	12.1 ± 0.5
Na^a^	0.679 ± 0.004	0.948 ± 0.002	0.639 ± 0.126	0.567 ± 0.041
Ni	< 5.82	< 146	< 77.6	< 179
Pb	< 69.0	< 86.1	< 45.9	< 106
Sr	565 ± 15	34.1 ± 1.2	409 ± 20	85.1 ± 3.8
V	4.89 ± 0.50	3.84 ± 0.24	1.16 ± 0.10	9.83 ± 0.93
Zn^a^	10.1 ± 0.1	11.1 ± 0.4	16.1 ± 0.01	2.31 ± 0.34

*VM* Vitamin and mineral, *BT* Botanical, *MN* Mineral

^a^Values in mg g^−1^

Samples selected for this work are intended to supplement nutritional deficiencies, containing a high concentration of essential elements in their composition (as presented in Table 1). This was confirmed by the results which showed a higher prevalence of essential elements such as Ca and Mg in all samples. Cobalt and Ni were the only essential elements with concentrations below the LOQ for all samples. The highest concentrations of Cu, Cr and Fe were observed in sample VM-II.

On the other hand, Al, Ba, Cr, Mo, and Sr were the non-essential elements with concentrations higher than the LOQ in all samples, although they were found at relatively low concentrations in some samples while higher for others. The samples classified as VM showed a higher content of elements when compared to the others due to their vitamin and mineral composition. The concentration of essential elements in samples of botanical (BT) origin may vary due to the type of plant it comes from, as well as whether any synthetic ingredients have been added to the product to achieve the expected level of supplementation. The non-essential elements were found to be present in low concentrations in all the samples. Aluminum was the element with the highest concentration in supplements of botanical origin.

Finally, concentrations of the essential and non-essential elements found in the food supplement samples were compared with those indicated on their label. Agreement values ranging from 80 to 120% were considered to be within acceptable limits. It is important to note that among the 14 elements found in all the samples, only a few elements—six elements for BT-I (Cu, Cr, Mg, Mn, Mo, and Zn) and for VM-I (Cu, Cr, Fe, Mn, Mo, and Zn) or eight elements for VM-II (Ca, Cu, Cr, Fe, Mg, Mn, Mo, and Zn) and for MN-I (Ca, Cu, Cr, K, Mg, Mn, V, and Zn)—have labeled values. Suitable agreements were obtained for Ca (110% in VM-II), Cu (98% in VM-I and 101% in MN-I), K (111% in MN-I), Mg (114% in VM-II and 94% in MN-I), and Zn (108% in MN-I). On the other hand, except for Zn (138%), the found concentration for the other elements in the sample BT-I showed an agreement lower than 3% with the labeled concentration. Slightly lower values were observed for Mg in VM-I (76%) and MN-I (75%), and for Zn in MN-I (74%), while somewhat higher agreements were obtained for Ca in VM-I (125%), Cu in VM-II (126%), Mg in VM-I (123%) and VM-II (123%), and Zn in VM-II (126%). Chromium and Mo exhibited an agreement ranging from 130 to 183% across all samples (except Mo in sample BT-I, as above mentioned). The observed discrepancies between the determined concentrations and the labeled values may be attributed to flaws during the food supplements manufacturing or quality control, and has also been observed in other studies (Krejčová et al. 2012; Latif et al. 2025). Furthermore, mislabeling can bring implications for the consumers, such as the unfulfillment of expected benefits or risks due to overexposure. However, it is important to underscore that, apart from those elements intentionally supplied at a higher dose, the concentration of the others does not exceed the dietary recommended intake (DRI) (Institute of Medicine 2006).

### Bioaccessibility assessment of essential and non-essential elements in food supplements

The variability of the bioaccessibility values of several elements comparing different bioaccessibility protocols has been previously observed, but there are few studies devoted to food matrices. Furthermore, it is noteworthy to note that most of these studies reported the bioaccessibility of only one or two elements in a single class of sample. Thus, an essential contribution of this work is the determination of bioaccessibility for a relatively large number of elements encompassing various types of food supplements.

The elements covered to evaluate the bioccessibility by UBM and INFOGEST were those that presented concentrations higher than the LOQ. Additionally, it was not possible to assess the bioaccessibility of Ca, K, and Na due to the high content of these elements in the reagents used to simulate the *in vitro* gastrointestinal digestion (as shown in Table 2). It is necessary to emphasize the relatively high LOQs obtained by both bioaccessibility methods (Table S3), especially for Mg, Sr, and Zn. Those high LOQs were expected due to the chemical composition of intestinal fluids, which drastically impaired the bioaccessibility assessment. The high carbon content in the digestive fluids can lead to both suppression and enhancement of analyte responses via different mechanisms (e.g., charge-transfer reactions and collisional ionization) (Serrano et al. 2021). Nevertheless, the expected high concentration of easily ionizable elements (EIEs) in the bioaccessible extracts may affect the excitation temperature and electron number density of the ICP source, thereby altering the ionization equilibrium (Wu and Hieftje 1994). In this sense, a minimum dilution factor was considered to minimize matrix interferences in measurements by ICP OES.

#### Determination of bioaccessibility using the UBM protocol

**Fig. 1:**
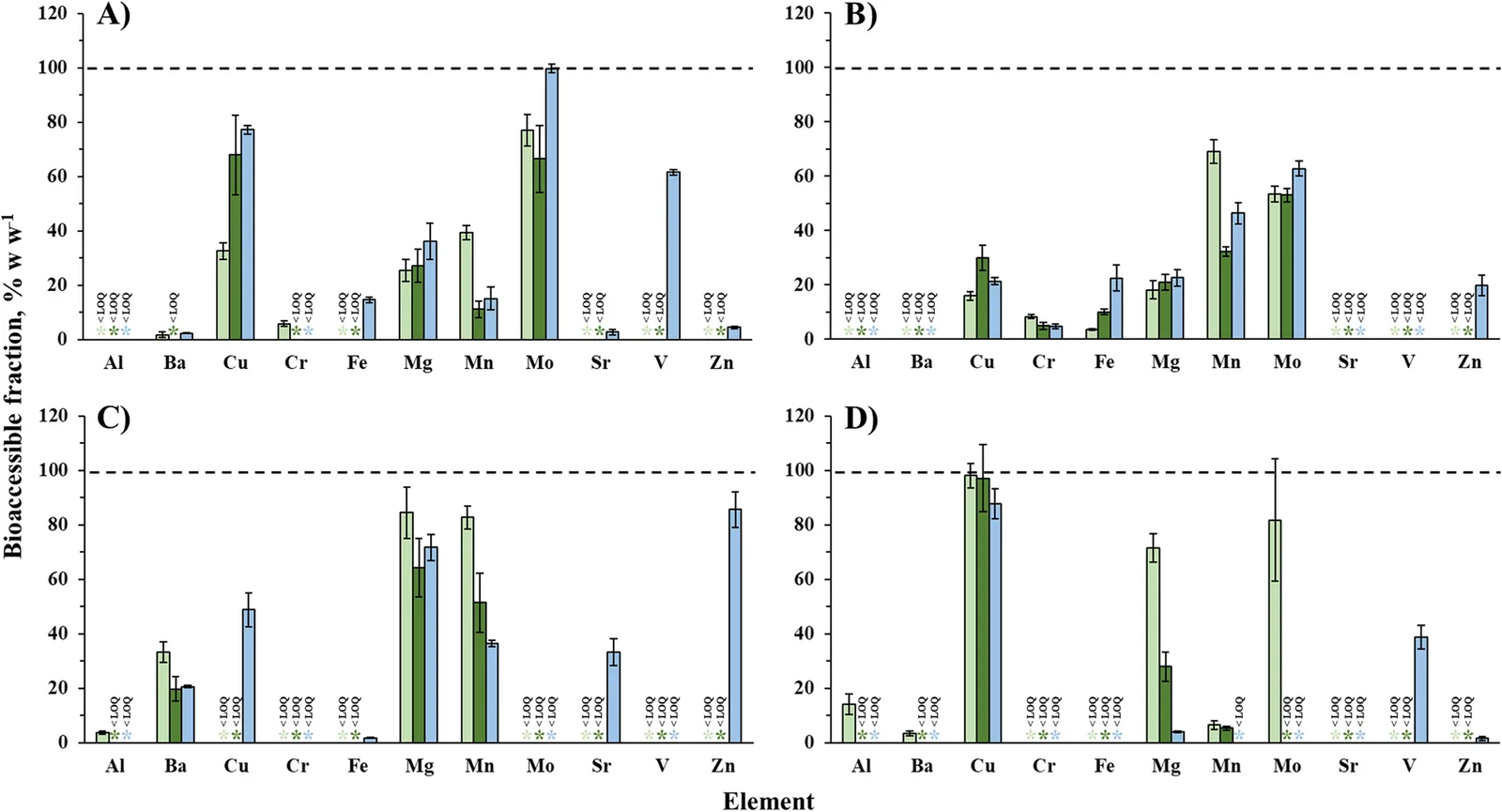
Bioaccessible fractions of elements in food supplements. **A)** VM-I; **B)** VM-II; **C)** BT-I; **D)** MN-I. The light green bar and dark green bar represent the results for the gastric and gastrointestinal phases of the UBM method, respectively, while the blue bar represents the results for the INFOGEST method. The dashed line represents the total concentration of the element in each sample (n = 5)

Considering the elements whose bioaccessibility evaluation was possible, values were calculated for both protocols. The bioaccessible fractions of the essential and non-essential elements in food supplements in the gastric (light green bars) and gastrointestinal (dark green bars) phases using the UBM protocol are shown in Fig. 1. In general, some metals had higher bioaccessibility in the gastric phase than in the gastrointestinal phase, which can be explained by better metal leaching at lower pH and pepsin complexation (Wragg et al. 2011). The lowest bioaccessibility fractions found in the gastric phase were for Al (4—14%), Ba (2—33%), Cr (6—8%), and Fe (4% for sample VM-II). The poor bioaccessibility of Cr can be a dependence on the chemical form and/or associated with antagonistic effects that emerge at high intakes of other metals, such as Fe, Zn, and Mn (Johnson et al. 1998). At the same time, the concentrations of Sr, V, and Zn were lower than LOQ in all samples and, thus the calculation of the bioaccessible fraction was not feasible.

Considering the gastrointestinal phase, Al, Sr, V, and Zn had concentrations below the LOQ for all samples, while Ba (20% for sample BT-I), Cr (5% for sample VM-II), and Fe (10% for sample VM-II) were the elements with the lowest bioaccessibility. This poor bioaccessibility of Al, Ba, Cr, Fe, Sr, V, and Zn in digestive fluids agrees with the literature. It is probably associated with the pH and the chemical environment of the intestinal fluids, as well as with the respective metallic species (Tokalioğlu et al. 2014; Cabrera-Vique and Mesías 2013; Bo 2000; Pelfrêne et al. 2017; Souza et al. 2021). Additionally, the higher LOQs for Sr and Zn in the gastric and gastrointestinal phases hinder their bioaccessible fraction assessment.

The highest bioaccessible fractions observed in both gastric and gastrointestinal phases by UBM method were for Cu (16–98% and 30–97%, respectively), Mg (18–84% and 21–64%) Mn (6–83% and 5–51%), and Mo (53–82% and 53–66%). This higher bioaccessibility also agrees with the previous reports (Tokalioğlu et al. 2014; Silva et al. 2018; Souza et al. 2021), where the authors usually correlate with their chemical forms (*e.g.*, Cu-amino acid and Mn-phytate complexes, present in sample MN-I (labeled) and BT-I (phytates are generally present in botanical matrices) (Silva et al. 2018; Bo 2000). Finally, the bioaccessible fractions found for Mg and Mo in samples VM-I and VM-II, as well as for Cu and Mn in sample MN-I were similar in the gastric and gastrointestinal phase, with no significant differences (*t-*test, confidence level of 95%).

#### Determination of the bioaccessibility using the INFOGEST method

The bioaccessible fractions of essential and non-essential elements in food supplements obtained using the INFOGEST method are also shown in Fig. 1 (blue bars). In general, the elements with the highest and the lowest bioaccessibility values were similar between the UBM and INFOGEST methods, as expected, since the chemical environment of the digestive fluids governs the bioaccessibility. The bioaccessible fraction of Al was lower than the LOQ for all samples. Barium (2–21%), Cr (5% for sample VM-II), Sr (3–33%), and Fe (2–22%) were the elements with the lowest bioaccessibility fraction determined by using the INFOGEST method. On the other hand, higher bioaccessible fractions were observed for Cu (21–88%), Mg (4–76%), Mn (15–46%), Mo (63–100%), and V (39–62%). Given that lower LOQs were obtained for Zn using the INFOGEST method, bioaccessible fractions ranging from 2 to 86% were obtained.

#### Comparison between the bioaccessible values assessed using the UBM and INFOGEST methods

No statistical differences (*t* test, confidence level of 95%) were observed between results from UBM (gastrointestinal phase) and INFOGEST protocols for Mg, Mn, and Cu in sample VM-I, Cr and Mg for samples VM-II, Ba and Mg in sample BT-I, and Cu in sample MN-I. On the other hand, significant differences appeared for Mo in sample VM-I, Cu, Fe, Mn, and Mo in sample VM-II, Mn in sample BT-I, and Mg in sample MN-I.

The results presented demonstrated that the bioaccessible fraction of elements is influenced by the bioaccessibility protocol employed. While a good agreement between methods was observed for certain elements, other ones showed discrepancies in the values. Generally, these differences were caused by a higher bioaccessible fraction by the INFOGEST method. As can be seen in Table S4, this protocol requires longer digestion time (4 h) compared to UBM method (2 h), as well as is performed under higher pH in all the phases, which might increase the bioaccessible fraction of some elements (even the leaching can be influenced by the pH). On the other hand, for elements which bioaccessible fraction was higher in the gastrointestinal phase of the UBM protocol (*i.e.*, Cu in sample VM-II, Mn in sample BT-I, and Mg in sample MN-I), variations in pH and bile salts/pancreatin concentration may be associated. For instance, Corte-Real et al. (2018) reported that Mg can form insoluble complexes with bile salts. Another important parameter that contrasts is the sample/liquid ratio (S/L) that means the amount of sample (g) in relation to the liquid (solution) volume (mL). Finally, it is important to note that the UBM method was initially designed for soil, whereas the INFOGEST was suggested for food samples and, thus, may be more suitable for food supplements.

Unfortunately, the lack of literature reporting a comparison of UBM and INFOGEST methods for these elements, since a single study has done it so far, but focused on As and Cd (Ma et al. 2024), as well as the absence of labeled information regarding the element species in some samples, make difficult to have a deep and consistent discussion of the results. However, the reported results corroborate the claim that modifications in the experimental parameters of invitro bioaccessibility protocols can lead to different responses and care must be taken when expressing and comparing results for different samples.

Considering that HCl is present in the fluids used in both bioaccessibility protocols, the extraction of analytes in an acidic medium, without the use of other reagents, was evaluated. Using the COST INFOGEST method, the extracted fractions were considered high for Mn (1–94%), Ba (2–79%), and V (5–74%), intermediate for Mo (2–68%), Mg (20–62%), Sr (35–61%), Cu (2–59%), Fe (57%), and Zn (1–48%), and low for Al (0.32–11%) and Cr (20%). Therefore, it is possible to consider that some elements are leached (some in high concentrations) using only the HCl solution.

## Conclusions

In this study, the bioaccessibility of essential and non-essential elements in food supplements following *in vitro* protocols was evaluated. The comparison between different bioaccessibility protocols (UBM and INFOGEST methods) revealed variability in the values. Particular attention must be devoted to the LOQs, which were relatively high, due to the composition of the extracts, which contain huge amounts of Ca, Na, and K. These high LOQs hindered the accurate assessment of bioaccessibility and comparison of protocols for some samples or elements. The comparison between the UBM and INFOGEST methods showed no statistical differences for certain elements, such as Ba, Cu, and Mg, but also revealed significant differences for other elements, including Fe and Mn. The lack of comparative literature between these methods and the complexity of extracts made a more in-depth analysis difficult, indicating the need for further research in this area. Attention should be given to the absence of information regarding the amino acid linked to the minerals limiting any deeper discussion on the related aspects. Furthermore, high-sensitivity techniques, such as ICP-MS, should be further applied to assess the total concentration and bioaccessibility fraction of elements present at lower concentration in food supplements, especially those considered toxic. Finally, the results obtained demonstrate the complexity of assessing bioaccessibility in different food supplements and highlight the need for improvement and standardization of methods for bioaccessibility assessment to ensure reliable results.

## Supplementary Information

Below is the link to the electronic supplementary material.

Supplementary file1 (DOCX 34 kb)

## Data Availability

Data will be made available on request.
