# Supplementary material for: Bioaccessibility of metals in food supplements: protocols and facts
Source: Biometals. 2026 Jun 30;39(5):1957–68. doi: 10.1007/s10534-026-00844-4 (PMC13518400; doi:10.1007/s10534-026-00844-4)
Supplement: Supplementary file 1 [file 10534_2026_844_MOESM1_ESM.docx]

# Bioaccessibility of metals in food supplements: protocols and facts

# Thaís dos Santos Berón, Gustavo Rossato Bitencourt, Rochele Sogari Picoloto, Rodrigo Cordeiro Bolzan, Paola Azevedo Mello*

Departamento de Química

Universidade Federal de Santa Maria

Av. Roraima, 1000, 97105-900

Santa Maria, RS, Brazil

# * Corresponding author. Tel: +55 55 3220 9445

Email address: [paola.mello@ufsm.br](mailto:paola.mello@ufsm.br)

**Supplementary Material**

# Table S1. Operational conditions for elemental determinations by ICP OES.

| **Parameter** | **ICP OES** |
| --- | --- |
| Radiofrequency power, W | 1400 |
| Main gas flow rate, L min ^-1^ | 15.0 |
| Auxiliary gas flow rate, L min ^-1^ | 0.2 |
| Nebulizer gas flow rate, L min ^-1^ | 0.7 |
| Nebulization chamber, type | Double-pass cyclonic (Scott-type) |
| Nebulizer, type | GemCone® |
| Replicates | 3 |
| Analytes | Emission line, nm |
| Ag | 328.068 |
| Al | 167.078 |
| Ba | 455.404 |
| Be | 234.861 |
| Cd | 228.802 |
| Co | 238.892 |
| Cr | 205.552 |
| Cu | 324.754 |
| Fe | 259.941 |
| La | 408.672 |
| Mg | 285.213 |
| Mn | 257.611 |
| Mo | 202.095 |
| Ni | 231.604 |
| Pb | 220.553 |
| Sr | 421.552 |
| V | 292.464 |
| Zn | 213.856 |

**Table S2**. Constituents of the digestive fluids used for BARGE and INFOGEST bioaccessibility protocols. Concentration between parenthesis (g L^-1^ unless otherwise specified).

| **BARGE** | | | | |  | **INFOGEST** | | |
| --- | --- | --- | --- | --- | --- | --- | --- | --- |
|  | Gastrointestinal phase | | | |  | Gastrointestinal phase | | |
|  | Gastric phase | |  | |  |  |  |  |
|  | **Salive** | **Gastric fluid** | **Duodenal fluid** | **Bile** |  | **Salive** | **Gastric fluid** | **Duodenal + bile fluid** |
| INORGANICS | KCl (0.9) | KCl (0.8) | KCl (0.6) | KCl (0.4) |  | KCl (1.1) | KCl (0.5) | KCl (0.5) |
|  | NaH_2_PO_4_ (0.9) | NaH_2_PO_4_ (0.3) | NaCl (7.0) | NaCl (5.0) |  | KH_2_PO_4_ (0.5) | KH_2_PO_4_ (0.1) | KH_2_PO_4_ (0.1) |
|  | KSCN (0.2) | NaCl (3.0) | NaHCO_3_ (6.0) | NaHCO_3_ (6.0) |  | NaHCO_3_ (1.1) | NaHCO_3_ (2.1) | NaHCO_3_ (7.1) |
|  | Na_2_SO_4_ (0.6) | CaCl_2_ (0.4) | CaCl_2_ (0.2) | CaCl_2_ (0.2) |  | MgCl_2_ (0.03) | NaCl (2.8) | NaCl (2.25) |
|  | NaCl (0.3) | NH_4_Cl (0.3) | KH_2_PO_4_ (0.08) | HCl (2 mmol L^-1^) |  | (NH_4_)_2_CO_3_ (0.006) | MgCl_2_ (0.02) | MgCl (0.07) |
|  |  |  | MgCl_2_ (0.05) |  |  | HCl (1.1 mmol L^-1^) | (NH_4_)_2_CO_3_ (0.05) | HCl (8.4 mmol L^-1^) |
|  | NaOH (0.07) | HCl (0.1 mol L^-1^) | HCl (2 mmol L^-1^) |  |  |  | HCl (15.6 mmol L^-1^) |  |
|  |  |  |  |  |  |  |  |  |
| ORGANICS | Urea (0.2) | Urea (0.085) | Urea (0.1) | Urea (0.25) |  |  |  |  |
|  | Uric acid (0.015) | Glucose (0.65) |  |  |  |  |  |  |
|  |  | Glucuronic acid (0.02) |  |  |  |  |  |  |
|  |  | Glucosamine (0.3) |  |  |  |  |  |  |
|  |  |  |  |  |  |  |  |  |
| ENZYMES | α-amylase (0.15) | Mucin (3.0) | Pancreatin (3.0) | Bile (6.0) |  | α-amylase (0.3) | Pepsin (1.75) | Pancreatin (1.1) |
|  | Mucin (0.05) | Pepsin (1.0) | Lipase (0.5) |  |  |  |  | Bile (27) |
|  |  | Bovine serum albumin (1.0) | Bovine serum albumin (1.0) | Bovine serum albumin (1.8) |  |  |  |  |
| pH | 6.5 ± 0.5 | 0.9-1.0 | 7.4 ± 0.2 | 8.0 ± 0.2 |  | 7.0 | 3.0 | 7.0 |
|  |  |  |  |  |  |  |  |  |

**Table S3**. Limits of quantification obtained for bioaccessibility solutions by ICP OES. Values between parenthesis represent the minimum dilution factor applied to mitigate matrix effects.

| **Element**  **(µg g^-1^)** | **BARGE method** | | **INFOGEST method** |
| --- | --- | --- | --- |
|  | **Gastric phase** | **Gastrointestinal phase** |  |
| Al | 8.88 (5) | 27.9 (5) | 3.08 (20) |
| Ba | 0.420 (n.a.) | 1.75 (n.a.) | 0.354 (5) |
| Cr | 0.972 (n.a.) | 2.44 (n.a.) | 3.57 (20) |
| Cu | 5.20 (n.a.) | 23.3 (n.a.) | 3.79 (5) |
| Fe | 24.7 (n.a.) | 83.0 (n.a.) | 5.00 (5) |
| Mg | 364 (n.a.) | 4521 (n.a.) | 188 (n.a.) |
| Mn | 3.95 (n.a.) | 11.9 (n.a.) | 1.54 (5) |
| Mo | 9.76 (5) | 18.9 (5) | 18.0 (20) |
| Sr | 115 (20) | 810 (20) | 4.04 (5) |
| V | 6.46 (n.a.) | 14.7 (n.a.) | 0.679 (n.a.) |
| Zn | 2720 (50) | 17612 (50) | 6.55 (5) |
| Sample mass, g | 0.3 | 0.3 | 5 |

n.a. = not applied.

**Table S4.** Comparison of UBM and INFOGEST parameters.

| **Phase** | **Parameter** | **UBM** | **INFOGEST** |
| --- | --- | --- | --- |
| **Oral** | pH | 6.5 ± 0.5 | 7 |
|  | Time, min | 1 | 2 |
|  | S/L* | 1:15 | 1:1 |
|  | α-amylase, g L^-1^ | 0.15 | 0.3 |
|  |  |  |  |
| **Gastric** | pH | 1.2 ± 0.05 | 3 |
|  | Time, h | 1 | 2 |
|  | S/L* | 1:37.5 | 1:3 |
|  | Pepsin (U mL^-1^) in total digest | 393 | 2000 |
|  |  |  |  |
| **Intestinal** | pH | 6.3 ± 0.5 | 7 |
|  | Time, h | 1 | 2 |
|  | S/L* | 1:97.5 | 1:7 |
|  | Bile salts, g L^-1^ | 0.9 | 14 |
|  | Pancreatin, g L^-1^ | 1.4 | 0.06 |

* S/L: sample (g) to liquid (mL) ratio
